# The Unique Contribution of Digital Maturity in the AI Era: Psychometric Validation, Predictive Ability and Latent Profile Analysis Among Chinese Primary Students

**DOI:** 10.3390/jintelligence14080172

**Published:** 2026-08-01

**Authors:** Meng Sun, Jiutong Luo

**Affiliations:** 1 College of Education for the Future, Beijing Normal University, Zhuhai 519087, China; msun@bnu.edu.cn; 2 Faculty of Education, Shenzhen University, Shenzhen 518060, China

**Keywords:** digital maturity, Chinese primary students, scale validation, predictive ability, latent profile analysis

## Abstract

With the widespread use of digital technologies, it has become increasingly important to understand how children adapt to digital environments. However, limited research has examined digital maturity among children beyond digital access and familiarity with technologies, particularly in non-Western contexts. Building on recent work on digital maturity, the present study aimed to validate the Digital Maturity Inventory (DIMI) among Chinese primary school students and to identify their latent profiles. A sample of 434 students (221 girls; Mean_age_ = 10.68, SD = 0.86) completed the DIMI for validation analyses, and an additional sample was simultaneously recruited, resulting in a total sample of 1026 students (500 girls; Mean_age_ = 10.38, SD = 1.09), for latent profile analysis. The findings demonstrated that the DIMI exhibited satisfactory psychometric properties in the Chinese context. Moreover, digital maturity, in addition to digital native, emerged as a unique and significant predictor of artificial intelligence (AI) literacy. Three distinct subgroups of students with heterogeneous patterns of digital maturity were also revealed: an unbalanced low group, an inconsistent medium group, and a relatively high group. These findings not only support the cross-cultural applicability of DIMI, but also demonstrate its relevance to children’s AI-related competencies. The implications of this study were also discussed.

## 1. Introduction

With the rapid advancement of digital technologies and the emergence of artificial intelligence (AI), young people are increasingly immersed in digital environments in their everyday lives (Chan & Lee, 2023; Hutton et al., 2024; OECD, 2025). Digital technologies bring significant opportunities, such as expanded access to information (Afzal et al., 2023; Warschauer & Matuchniak, 2010), new forms of social interaction (Canale et al., 2022; Yıldız & Nur, 2024), and innovative educational experiences (Blau & Shamir-Inbal, 2018; Oliveira & De Souza, 2022). In particular, AI, including both discriminative and generative nature (e.g., Zheng et al., 2023), has further enriched young people’s digital experience with intelligent and prompt feedback (Yan et al., 2024; Zhu et al., 2023). At the same time, potential negative consequences of digital technology use have been raised, including distraction (Dontre, 2021; Luo et al., 2025a), overuse (Büchi et al., 2019; Montag & Elhai, 2020), and risks to mental health and well-being (Hutton et al., 2024; Orben & Przybylski, 2019).

The concepts for digital generations have also been evolving rapidly. The most well-known is from Prensky (2001), who coined the notion of digital immigrant and digital native. Digital immigrants refer to the generation who grew up along with the introduction of personal computers, early mobile access, and the Internet, when the world was turning digital gradually (Prensky, 2001). However, digital natives mostly refer to those who were born after the 2000s (i.e., millennials) who grew up during the rapid expansion of the Internet. For the current context, people who are truly digital-born have access to various digital devices, such as smartphones, tablets, etc., as a central part of their growing experience. According to Prensky (2001), most children are now digital natives. Previously, Teo (2013) developed and validated a digital native assessment scale among a sample of Australian high school students, and this scale has been applied to many cultural contexts (Huang et al., 2021; Kesharwani, 2020; Teo, 2016).

The concept of digital natives has been widely used to describe young generations’ familiarity and exposure to digital technologies (Teo, 2013); however, it primarily reflects demographic characteristics and technological experiences. Being born into a digitized environment does not necessarily guarantee the ability to critically evaluate, regulate, and use digital technologies responsibly. Therefore, digital maturity, a conceptually distinct construct emphasizing developmental readiness and adaptive capacities for navigating digital environments, has been proposed (Laaber et al., 2023). Specifically, grounded in psychosocial maturity and self-determination theory (Bleidorn, 2015; Deci & Ryan, 2000; Greenberger et al., 1975; Wehmeyer et al., 2017), digital maturity included three capacities and ten sub-dimensions (for details, see Laaber et al., 2023). Compared with digital literacy, which primarily focuses on the knowledge and skills required to effectively use digital technologies (e.g., Churchill, 2020), digital maturity represents a broader developmental perspective by incorporating motivational, regulatory, and responsible dimensions of digital engagement (Laaber et al., 2023). In fact, digital maturity may be understood as an adaptive manifestation of intelligence that emerges through individuals’ continuous interaction with digital technologies and develops through practice in technology-rich environments.

While digital maturity has its potential in the rapid penetration of digital technologies, empirical research on digital maturity is still emerging and has so far been concentrated in European contexts (Christensen et al., 2025; Hofmans et al., 2024; Koch et al., 2025; Laaber et al., 2023, 2024). For example, Laaber et al. (2023) conceptualized, developed, and validated the DIMI among adolescents in Germany and Austria, establishing its factor structure, reliability, and incremental validity to problematic mobile use. Koch et al. (2025) further demonstrated its predictive relevance by linking digital maturity to social connectedness and mechanisms of online well-being. Hofmans et al. (2024) applied a psychometric network approach, revealing central domains such as support-seeking and impulse regulation.

Despite these advances, several important research gaps remain. First, existing validation studies have been conducted primarily in Western samples (e.g., Austria, Germany, and The Netherlands), with limited evidence regarding whether digital maturity represents a culturally generalizable developmental construct among children. This limitation is particularly important in the Chinese context, where children’s digital engagement has increased rapidly in recent years. According to the CNNIC reports (CNNIC, 2021, 2024), Internet access among Chinese children increased from 89.5% in 2018 to 95.1% in 2022 and further reached 97.2% in 2023. Therefore, examining the psychometric properties and applicability of digital maturity among Chinese primary students can extend current understanding of children’s digital development across cultural contexts. Furthermore, although digital maturity emphasizes adaptive, self-regulatory, and responsible engagement with digital technologies, its relevance to emerging AI-related competencies remains insufficiently understood. Specifically, it remains unclear whether digital maturity explains unique differences in AI literacy beyond technology exposure and demographic characteristics.

Second, prior studies have mainly adopted variable-centered approaches (e.g., factor analysis, regression, network models, and machine learning) to examine digital maturity. Although these approaches provide valuable insights into general relationships among variables, they may overlook qualitative differences in how children develop and engage with digital technologies. A person-centered approach, such as latent profile analysis, can provide additional insights by identifying distinct patterns of digital maturity and revealing heterogeneous developmental needs among children. Understanding such patterns may further inform differentiated educational practices for supporting children’s digital development.

In this case, this study aimed to extend the understanding of digital maturity among Chinese primary students by conducting two complementary studies. Study 1 focused on validating the digital maturity inventory (DIMI) among Chinese primary students. Beyond examining the construct validity and reliability, this study also tested discriminant validity with the digital native assessment scale and predictive validity of digital maturity for artificial intelligence (AI) literacy—an emerging and critical concept in the AI era. In fact, the emergence of AI technologies further highlights the importance of digital maturity beyond basic digital familiarity. Thus, AI literacy was selected as the criterion variable because AI represents a transformative extension of contemporary digital environments. Unlike general digital competence, which mainly concerns the effective use of existing digital tools, AI literacy requires individuals to understand, evaluate, and interact with intelligent systems in more complex and dynamic contexts. Therefore, AI literacy provides a timely indicator for examining whether students’ digital maturity prepares them for emerging AI changes. Previous studies have suggested that students’ digital skills and technology-related self-efficacy provide important foundations for developing AI-related knowledge and skills (e.g., Chai et al., 2024; Luo et al., 2025b; Ng et al., 2021). Moreover, effective AI use requires not only technical familiarity but also self-regulation, critical evaluation, and responsible technology practices, which are closely related to the dimensions of digital maturity (Laaber et al., 2023). Therefore, examining whether digital maturity relates to AI literacy may provide new insights into children’s readiness for the AI era. In addition, Study 2 employed an exploratory latent profile analysis (LPA) to uncover potential subgroups of primary students characterized by different patterns of digital maturity, thereby providing a more nuanced understanding of individual differences in digital maturity and offering implications for cultivating young generations’ digital maturity. Given the psychometric validation and exploratory person-centered objectives of the two studies, the present research was guided by research questions rather than directional hypotheses. Three research questions (RQs) were addressed by the current study:

RQ1: Does the Digital Maturity Inventory demonstrate satisfactory psychometric properties, including factorial validity and reliability, among Chinese primary students?

RQ2: Does digital maturity significantly relate to students’ AI literacy?

RQ3: What latent profiles of digital maturity emerge among Chinese primary students?

## 2. Study 1: Validation of the Digital Maturity Scale Among Chinese Primary Students

### 2.1. Methods

#### 2.1.1. Participants

Study 1 recruited 459 students from a public primary school in southern China using convenience sampling. To ensure data quality, two attention-check items requiring students to select “always” were included to identify potentially invalid responses. Questionnaires with failed attention checks were excluded from further analysis. No missing data were observed among the retained responses. Finally, 434 students (94.6% valid response rate; 221 girls; Mean_age_ = 10.68, SD = 0.86) were retained for further analysis. All participants were asked to finish an online questionnaire at school, which took about 15 min. Informed consent was obtained from parents and schools, and all participants signed the consent form and were informed that they could withdraw from the study at any time without any negative consequences. To ensure data privacy and confidentiality, all responses were anonymized before analysis. The collected data were securely stored and were accessible only to the research team for research purposes. This study was conducted in accordance with the ethical guidelines of the authors’ university. In addition, students were also asked to report their average digital device use time on weekdays and weekends, respectively. The details of the demographic information are shown in Table 1.

#### 2.1.2. Measures

**Digital maturity inventory:** The 32-item digital maturity inventory (DIMI) was adopted (Laaber et al., 2023). In order to validate the DIMI in the Chinese context, we followed the forward and backward translation procedure. First, an expert in children’s media studies with bilingual proficiency independently translated the original English items into Chinese. In addition, ChatGPT-4o was used as an auxiliary linguistic tool to generate an initial translation draft, which was not directly adopted but served as a reference during the translation process. The expert researcher reviewed and refined the AI-generated suggestions. The two translation versions were then compared and synthesized into a preliminary Chinese version. Next, another bilingual researcher who was blinded to the original English version back-translated the preliminary Chinese version into English. Any discrepancies between the back-translation and the original items were carefully reviewed and resolved through discussion among the researchers. The researchers involved in the translation and review process had expertise in children’s media studies and educational technology, ensuring that the adapted items remained consistent with the original construct while being appropriate for the Chinese context. Finally, the preliminary version was piloted with primary students to examine item clarity and comprehensibility. Although formal cognitive interviews were not conducted, students’ feedback regarding item understanding was considered during the final refinement of the scale. The detailed validation process was discussed in the following sections.

**Digital native assessment scale:** This scale was adopted from Teo’s (2013) work. Originally, the scale contained 21-items with four dimensions. To make the overall questionnaire as short as possible and fit the primary students, we adopted 12 items, with 3 items for each dimension. Two pre-service teachers with primary school teaching experience reviewed the items, and a pilot test was conducted to ensure that students could clearly understand the items. The overall Cronbach’s alpha for this scale was 0.92, and the Cronbach’s alpha(s) for each dimension ranged from 0.79 to 0.88, which were also acceptable.

**AI literacy scale:** The AI literacy scale was adopted from Zhong and Liu (2025). This scale was originally developed for Chinese secondary students. Similarly, the same two pre-service teachers reviewed the items and selected 7 items from the AI knowledge and affective dimensions to fit the primary students (e.g., “I can describe the meaning and characteristics of AI”). The Cronbach’s alpha for the selected items in this study was 0.92 in this study.

#### 2.1.3. Data Analysis

The data analysis included several steps. At the beginning, the collected data was input into SPSS 27.0 for analysis. Three dimensions (i.e., autonomous choice to use mobile devices, regulation of negative emotions in digital contexts, and regulation of impulses in digital contexts) were reverse-coded accordingly (Laaber et al., 2023). Then, descriptive statistics were computed for all digital maturity variables, followed by correlational analyses to examine their interrelationships. Meanwhile, the internal consistency was assessed with Cronbach’s alpha. Second, construct validity was evaluated through confirmatory factor analysis (CFA) using maximum likelihood estimation in Amos 24.0. Following Laaber et al. (2023), the overall digital maturity score was computed as a weighted composite score of the ten dimensions rather than modeled as a second-order latent factor. Thus, only first-order CFA was performed on the dataset. Model fit was determined through multiple indices, including χ^2^/df, which should be between 1 and 3, comparative fit index (CFI) and Tucker–Lewis index (TLI) both above 0.90, and root mean square error of approximation (RMSEA) should be below 0.08 (Hu & Bentler, 1999). Third, to evaluate the discriminant validity, the correlations among the overall digital maturity scale and digital natives (including its sub-dimensions) were examined. Last but not least, the predictive validity was preliminarily assessed through a hierarchical, block-wise regression with three steps to determine the extent to which digital maturity predicted AI literacy, which is an emerging literacy for future students.

### 2.2. Results

#### 2.2.1. Results for Descriptive, Correlational, and Reliability Analyses for the Digital Maturity Inventory

Table 2 shows the descriptive, correlational, and reliability statistics for ten sub-dimensions of the digital maturity inventory. Specifically, the Cronbach’s alpha ranged from 0.70 to 0.91, indicating an acceptable to excellent level of internal consistency for the Chinese version of the digital maturity inventory. The correlations among the ten sub-dimensions of the digital maturity inventory were also shown.

#### 2.2.2. Results for Validity Analysis

As the main objective of this study was to validate the digital maturity scale among the Chinese primary students, we conducted the confirmatory factor analysis (CFA) with Amos 24.0. Following the original conceptualization study (Laaber et al., 2023), in which overall digital maturity is modeled as a weighted linear combination of ten sub-dimensions, we only conducted the first-order CFA to validate the scale. The model fit was acceptable: χ^2^/df = 1.988, *p* = 0.000, CFI = 0.948, TLI = 0.938, RMSEA = 0.048, 90% CI [0.043, 0.053] in this study. Table 3 shows the factor loading, composite reliability (CR), and average variance extracted (AVE) for the Chinese version scale. The CRs exceeded 0.70, and AVEs ranged from 0.45 to 0.78. Although the AVE for the autonomous choice to use mobile devices (DM1) was slightly below the recommended threshold (0.50), it was still considered acceptable according to the previous literature (Fornell & Larcker, 1981; Muhamad Safiih & Azreen, 2016), especially given that all factor loadings with this dimension were above 0.50. Meanwhile, although relatively strong correlations were observed between some dimensions (i.e., DM4 and DM5, DM7 and DM8), the discriminant validity of the constructs was still supported. As shown in Table 2, the square roots of the AVEs for all dimensions were higher than their correlations with other constructs, indicating that each dimension shared more variance with its own indicators than with other dimensions (Fornell & Larcker, 1981; Luo et al., 2025c). Therefore, the observed correlations among these dimensions reflected their conceptual relatedness rather than a lack of construct distinctiveness.

After confirming the construct validity of the Chinese version digital maturity inventory, we further computed the weighted digital maturity score according to the conceptualization work (Laaber et al., 2023), i.e., DM = (9.91 × DM1 + 9.39 × DM2 + 10.17 × DM3 + 10.43 × DM4 + 11.73 × DM5 + 9.39 × DM6 + 10.30 × DM7 + 9.91 × DM8 + 10.30 × DM9 + 8.47 × DM10)/100, and analyzed its correlations with digital natives and its sub-dimensions (see Table 4). The results indicated that digital maturity significantly but only moderately (Cohen, 1988) correlated with digital natives (r = 0.25, *p* < 0.001), and its sub-dimensions, with the smallest for grow up with technology (r = 0.10, *p* < 0.05) and the highest for thrive on instant gratifications and rewards (r = 0.30, *p* < 0.001).

For the predictive validity, this study adopted a new and important concept, that is, artificial intelligence (AI) literacy, the results shown in Table 5 indicating that, after controlling gender, grade, time spent on digital devices during weekdays and weekends, the digital maturity contribute uniquely to AI literacy, i.e., B = 0.559 95% CI [0.397, 0.722], β = 0.285, *t* = 6.764. Importantly, digital maturity explained an additional 7.5% of the variance in AI literacy (ΔR^2^ = 0.075), suggesting a meaningful contribution beyond demographic (gender and grade), time spent on both weekdays and weekends, as well as digital native.

### 2.3. Discussion

The findings of Study 1 provide support for the applicability of the digital maturity construct and its measurement among Chinese primary school students. The results demonstrated acceptable reliability and validity of the scale in this context, thereby extending prior research conducted in Western settings (Christensen et al., 2025; Hofmans et al., 2024; Koch et al., 2025; Laaber et al., 2023, 2024). In addition, digital maturity was slightly to moderately correlated with digital native and its sub-dimensions (r = 0.10–0.30; Cohen, 1988). In particular, the relationship between digital maturity and growing up with technology was the smallest, whereas the relationship between digital maturity and thriving on instant gratifications and rewards was the highest. This result might be in accordance with the fact that most Chinese primary students have already grown up with digital technologies (CNNIC, 2021, 2024), and further indicated that digital maturity had captured a specific capabilities and attitudes of individual growth and social adjustment (Laaber et al., 2023), which might be more related to the resistance of instant gratifications and rewards from digital technologies (Teo, 2013). Finally, both digital maturity and digital native were found to be significant predictors of students’ AI literacy. Moreover, digital maturity explained unique variance in AI literacy beyond that accounted for by digital native, age, gender, and time spent on digital devices during weekdays and weekends, highlighting its distinctive contribution in the AI era. Consistent with previous studies (e.g., Chai et al., 2024; Luo et al., 2025b; Ng et al., 2021), the present findings further highlight digital maturity as an important developmental foundation associated with AI literacy. Taken together, these results underscore the value of digital maturity as a construct that captures deeper aspects of students’ growth and adaptive functioning in the digital age (Laaber et al., 2024), beyond mere familiarity with technology (Prensky, 2001; Teo, 2013). Especially, the results further suggest that digital maturity represents an essential competency for preparing young children to thrive in the digital and AI era.

## 3. Study 2: Latent Profiles Analysis Regarding Digital Maturity Among Chinese Primary Students

### 3.1. Methods

#### 3.1.1. Participants

In Study 2, we recruited an additional 592 primary students from another primary school in southern China within the same district using convenience sampling. Similar to Study 1, the original sample consisted of 628 students, among whom those failing the attention checks were excluded (valid response rate = 94.3%). The data collection for Study 1 and Study 2 was conducted during the same period, and both samples were recruited from primary schools in southern China using convenience sampling. The two datasets shared the same measurement of digital maturity, while Study 1 additionally included the assessment of AI literacy for examining predictive validity. Therefore, the two datasets were combined to provide a larger sample for the exploratory latent profile analysis. The combined sample consisted of 1026 students (500 girls; Mean_age_ = 10.38, SD = 1.09). The overall details of the demographic information were also shown in Table 1. Although the two samples differed somewhat in grade distributions due to school-based sampling characteristics (e.g., the absence of third graders in Study 1 and variations in the proportions of students across Grades 4–6), they were recruited from comparable primary school contexts in southern China and used the same measurement framework for digital maturity. Nevertheless, the potential influence of institutional and grade-related differences on the identified latent profiles should be acknowledged as a limitation.

#### 3.1.2. Measures

**Digital maturity inventory:** As in Study 1, we adopted the validated Chinese version of the digital maturity inventory to measure primary students’ digital maturity (Laaber et al., 2023). The overall Cronbach’s alpha was 0.85, and the Cronbach’s alpha for each dimension ranged from 0.69 to 0.90, indicating an acceptable reliability in a social science study.

#### 3.1.3. Data Analysis

The data were input into SPSS 27.0 for analysis. First, the descriptive, correlational and reliability analyses were conducted. Second, the latent profile analysis was performed with Mplus 8.0 to identify the subgroups. The number of profiles was determined by multiple model indices such as the Akaike Information Criterion (AIC), Bayesian Information Criterion (BIC), adjusted BIC (aBIC), entropy, *p*-value of the Lo-Mendell-Rubin likelihood ratio test (LMR), and bootstrapping likelihood ratio test (BLRT; Geiser, 2013; Ram & Grimm, 2009). Finally, ANOVAs were also performed to compare the differences between each subgroup on the overall and ten sub-dimensions of digital maturity.

### 3.2. Results

Table 6 shows the descriptive, correlational, and reliability statistics for the ten sub-dimensions of the digital maturity inventory. Specifically, the Cronbach’s alpha ranged from 0.69 to 0.90, which further confirmed the acceptability of internal consistency for the Chinese version digital maturity inventory. The correlations among the ten sub-dimensions of digital maturity inventory were also shown.

#### The Latent Profiles

As shown in Table 7, among the tested solutions, the three-profile model was selected as the optimal solution based on a combination of statistical fit indices, classification quality, and substantive interpretability. Although the AIC, BIC, and aBIC values continued to decrease with an increasing number of profiles, the three-profile solution yielded the highest entropy value (i.e., 0.896), indicating excellent classification accuracy. In addition, the LMR and BLRT tests were significant for the three-profile solution, suggesting that adding the third profile significantly improved model fit compared with the two-profile solution.

**Figure 1:**
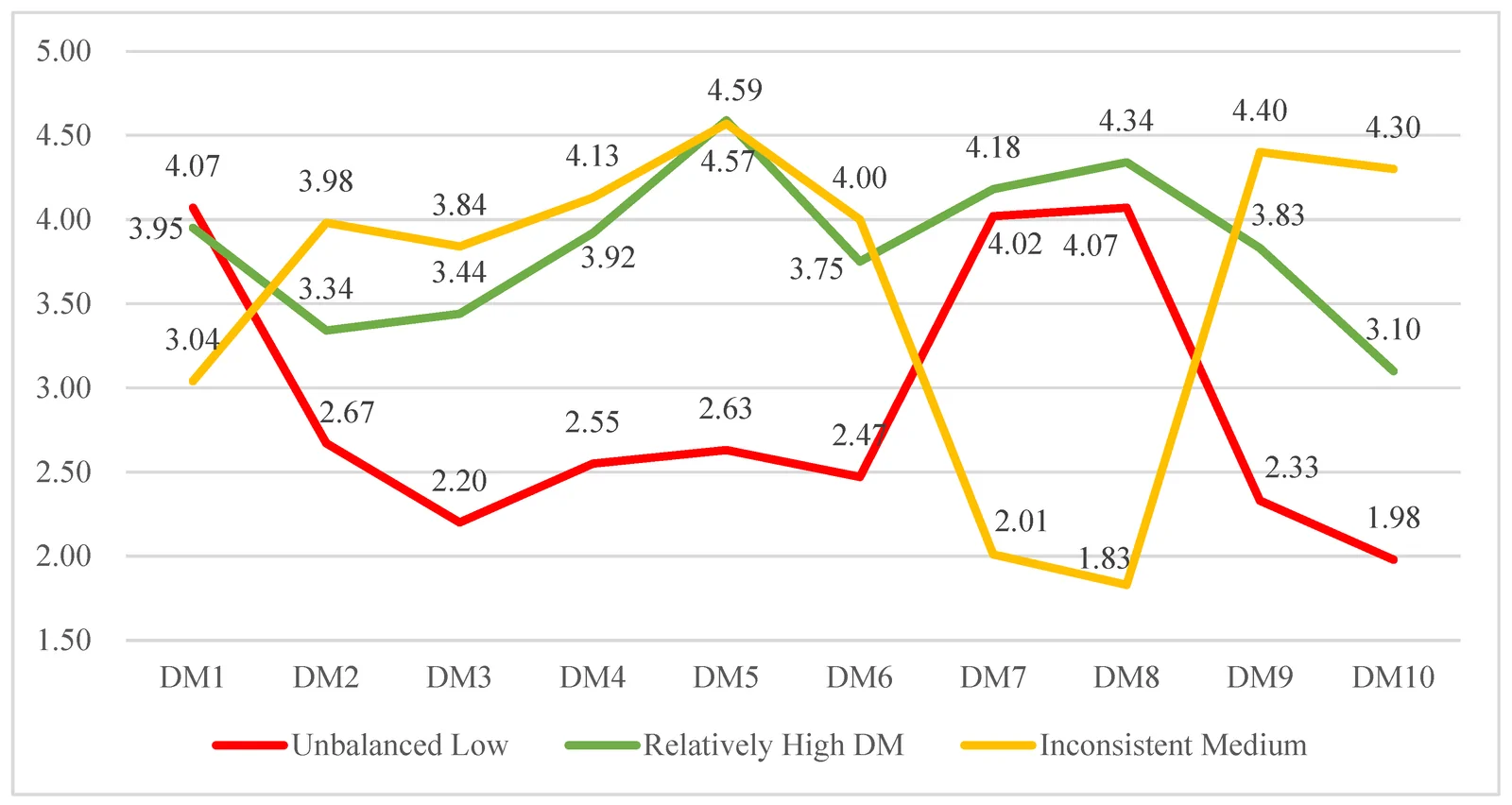
The mean scores of ten dimensions in the two digital maturity profiles. Note. DM1 = Autonomous Choice to Use Mobile Devices; DM2 = Autonomy Within Digital Contexts; DM3 = Digital Literacy; DM4 = Individual Growth in Digital Contexts; DM5 = Digital Risk Awareness; DM6 = Support-Seeking Regarding Digital Problems; DM7 = Regulation of Negative Emotions in Digital Contexts; DM8 = Regulation of Impulses in Digital Contexts; DM9 = Respect Towards Others in Digital Contexts; DM10 = Digital Citizenship.

Although the four-profile solution also demonstrated acceptable statistical fit, it was not selected because the additional profile did not provide a substantively distinct and theoretically meaningful pattern beyond the three-profile solution. Moreover, the four-profile solution resulted in a relatively small subgroup, with the smallest profile consisting of approximately 78 participants (7.6% of the total sample), which may affect the stability and interpretability of the identified profile. Therefore, considering both statistical evidence and theoretical interpretability, the three-profile model was retained as the final solution. Finally, profiles 1, 2, and 3 encompassed 21.0% (n = 215), 68.6% (n = 704), and 10.4% (n = 107) samples, respectively. Figure 1 illustrates the mean scores for ten sub-dimensions of digital maturity across the profiles, and Table 8 demonstrates the results from ANOVAs.

Profile 1 exhibited relatively high scores only on autonomous choice to use mobile devices (DM1), regulation of negative emotions (DM7), and regulation of impulses (DM8), but ranked lowest overall in overall digital maturity among the three groups. Thus, this profile was labeled the unbalanced low group.

Profile 3 occupied the middle position in overall digital maturity but displayed an inconsistent pattern. Specifically, this group scored highest on autonomy within digital contexts (DM2), digital literacy (DM3), individual growth in digital contexts (DM4), support-seeking regarding digital problems (DM6), respect toward others in digital contexts (DM9), and digital citizenship (DM10), while showing extremely low scores on DM1, DM7, and DM8. Accordingly, this profile was named the inconsistent medium group.

In terms of Profile 2, the largest subgroup, showed the highest overall level of digital maturity. Compared with Profile 3, it obtained lower scores on most sub-dimensions, but stood out by achieving the highest levels on both regulation of negative emotions (DM7) and regulation of impulses (DM8). Importantly, however, the mean digital maturity score of this group was 3.87, indicating a relatively higher level of digital maturity compared with the other profiles rather than an absolute optimal level. Therefore, this subgroup was labeled the relatively high group.

### 3.3. Discussion

The latent profile analysis revealed three distinct subgroups of primary school students, each characterized by different patterns of digital maturity. The first subgroup, labeled the unbalanced low group, showed overall low levels of digital maturity. This profile may represent children who experience relatively autonomous digital engagement but may lack sufficient external guidance. Greater autonomy may provide them with more opportunities to encounter diverse digital situations and may indicate more emotion and impulse regulations; however, their development in other sub-dimensions of digital maturity seems to may depend on external support (Deci & Ryan, 2000; Bernier et al., 2010). In this case, parental modeling and active mediations may serve as important support for children’s digital media use (e.g., Luo et al., 2023).

The second subgroup, the inconsistent medium group, demonstrated a mixed profile. This pattern may reflect a reliance on external sources of control—such as parents or teachers—to regulate digital technology use and negative emotions (Lee, 2013; Valkenburg et al., 1999), which can provide short-term protection but may hinder the development of internalized self-regulation (Deci & Ryan, 2000).

The largest subgroup, labeled the relatively high group, demonstrated the most favorable overall profile. Although their mean score indicated room for improvement, this group suggests that autonomy-supportive digital environments may be associated with more favorable patterns of digital maturity. Research showed that autonomy-supportive environments were crucial for developing self-regulation and adaptive competencies in digital settings (e.g., Morales-Álvarez et al., 2025). These findings tentatively suggest that allowing children to make relatively autonomous choices and take responsibility for their digital engagement, even if this entails certain trade-offs in other sub-dimensions of digital maturity, might correlate with deeper growth and adjustment in the digital and AI era. Given that the latent profiles were identified from a single sample, these interpretations should be considered preliminary and warrant further investigation.

## 4. General Discussion

Several limitations should also be noted. First, although the present study provided initial evidence supporting the validity and reliability of the Chinese version of the DIMI, further validation is still needed. In particular, future studies should examine alternative measurement models (such as a bifactor model) and further conduct measurement invariance analyses across various cultural contexts, educational levels, and demographic groups (such as gender and grade) using comparable samples. Such investigations would provide stronger evidence regarding the cross-cultural applicability and generalizability of the DIMI. Meanwhile, although the present study provided initial evidence regarding the predictive validity of digital maturity for AI literacy, future research could employ more rigorous analytical approaches, such as structural equation modeling, to further examine the relationships between digital maturity and other relevant external criteria. Second, although AI literacy was included as an important criterion variable in this study, it was assessed using a relatively brief adapted measure. In addition, the digital native assessment scale was also shortened to reduce the response burden for primary students in our sample. Future research should incorporate more comprehensive measures to further examine the relationship between digital maturity and related variables. Third, the sample was limited to primary school students from a single city in China, which restricted the generalizability of the findings. Moreover, although Study 1 and Study 2 were conducted within comparable educational contexts, the two samples differed somewhat in grade distributions and school contexts due to school-based convenience sampling. Such sample heterogeneity may have influenced the identified patterns of digital maturity. In addition, the participating schools may not fully capture the diversity of Chinese primary schools in terms of regional characteristics, educational resources, and students’ socioeconomic backgrounds, which were not directly assessed in the current study. Future research should recruit larger and more diverse samples to replicate and extend the results. Fourth, the latent profile analysis revealed three meaningful subgroups of students; however, the sizes of the identified profiles were relatively unequal, with some profiles containing fewer participants. Therefore, the identified profiles should be interpreted cautiously and further validated in independent samples. Fifth, the cross-sectional design precluded any causal inferences. Longitudinal and experimental studies are needed to determine whether enhancing digital maturity leads to improvements in AI literacy and broader developmental outcomes. Finally, all data were collected through self-report surveys, which may be influenced by social desirability or students’ limited self-awareness. Future studies might incorporate multiple informants (e.g., parents or teachers), behavioral observations (e.g., actual device use time), and interviews to triangulate the data.

Overall, the present research preliminarily validated the digital maturity inventory among Chinese primary school students and examined its relevance in the digital and AI era. Study 1 confirmed that the Chinese version of the scale could be a reliable and valid measure, and that digital maturity uniquely relates to AI literacy beyond the influence of digital native. Study 2 further identified three distinct profiles of digital maturity (i.e., imbalanced low, inconsistent medium, and relatively high) from a convenience sample. Taken together, within the current sample, these findings indicate that digital maturity among Chinese primary students is heterogeneous rather than uniform. These findings have important implications for educational practices and policies. Specifically, the validated digital maturity inventory might serve as a useful tool for identifying students’ developmental needs, while the identified profiles highlight the importance of differentiated digital education that moves beyond technical skill training and promotes autonomy, self-regulation, responsible digital engagement, and adaptive capacities in digital and AI-related contexts.

## Figures and Tables

**Table 1:** The demographic information of the participants for Study 1 and Study 2.

Variables	Items	Study 1 Frequency (N)	Ratio (%)	Study 2 Frequency (N)	Ratio (%)
Gender	Boys	213	49.1%	526	51.3%
	Girls	221	50.9%	500	48.7%
Grade	Grade 3	NA	NA	112	10.9%
	Grade 4	94	21.7%	326	31.8%
	Grade 5	238	54.8%	312	30.4%
	Grade 6	102	23.5%	276	26.9%
Time spent on weekdays	Less than 0.5 h	157	36.2%	253	24.7%
	0.5–1 h	123	28.3%	305	29.7%
	1–2 h	77	17.7%	232	22.6%
	2–3 h	33	7.6%	122	11.9%
	3–4 h	17	3.9%	61	5.9%
	Above 4 h	27	6.2%	53	5.2%
Time spent on weekends	Less than 0.5 h	97	22.4%	140	13.6%
	0.5–1 h	115	26.5%	206	20.1%
	1–2 h	101	23.3%	245	23.9%
	2–3 h	53	12.2%	194	18.9%
	3–4 h	24	5.5%	124	12.1%
	Above 4 h	44	10.1%	117	11.4%

**Table 2:** Reliability statistics and correlations among the digital maturity sub-domains for Study 1.

Variables	1	2	3	4	5	6	7	8	9	10	Cronbach’s Alpha
DM1 ^a^	0.67										0.70
DM2	−0.54 ***	0.81									0.85
DM3	−0.29 ***	0.39 ***	0.73								0.76
DM4	−0.22 ***	0.36 ***	0.49 ***	0.78							0.82
DM5	−0.07	0.25 ***	0.35 ***	0.64 ***	0.88						0.91
DM6	−0.10 *	0.18 ***	0.25 ***	0.49 ***	0.56 ***	0.81					0.87
DM7 ^a^	0.44 ***	−0.26 ***	−0.19 ***	−0.14 **	−0.07	−0.24 ***	0.78				0.81
DM8 ^a^	0.45 ***	−0.30 ***	−0.18 ***	−0.11 *	−0.01	−0.11 *	0.69 ***	0.83			0.87
DM9	−0.14 **	0.27 ***	0.26 ***	0.39 ***	0.50 ***	0.38 ***	−0.17 ***	−0.22 ***	0.76		0.84
DM10	−0.20 ***	0.22 ***	0.42 ***	0.41 ***	0.36 ***	0.32 ***	−0.34 ***	−0.34 ***	0.59 ***	0.84	0.87
Mean (SD)	3.78 (0.92)	3.25 (1.16)	3.19 (1.07)	3.70 (0.99)	4.23 (1.01)	3.58 (1.12)	3.86 (1.09)	3.89 (1.21)	3.66 (1.12)	3.02 (1.30)	

Note: * *p* < 0.05; ** *p* < 0.01; *** *p* < 0.001. ^a^ According to the original digital maturity scale, items for DM1, DM7, and DM8 were reversed coded and these sub-domains were calculated after revise coding. DM1 = Autonomous Choice to Use Mobile Devices; DM2 = Autonomy Within Digital Contexts; DM3 = Digital Literacy; DM4 = Individual Growth in Digital Contexts; DM5 = Digital Risk Awareness; DM6 = Support-Seeking Regarding Digital Problems; DM7 = Regulation of Negative Emotions in Digital Contexts; DM8 = Regulation of Impulses in Digital Contexts; DM9 = Respect Towards Others in Digital Contexts; DM10 = Digital Citizenship. Diagonal values are square root of AVEs of constructs.

**Table 3:** CFA results.

Variables	Items	Factor Loading	CR	AVE
DM1	DM1_01	0.68	0.71	0.45
	DM1_02	0.73		
	DM1_03	0.58		
DM2	DM2_01	0.72	0.85	0.66
	DM2_02	0.84		
	DM2_03	0.86		
DM3	DM3_01	0.67	0.77	0.53
	DM3_02	0.82		
	DM3_03	0.69		
DM4	DM4_01	0.81	0.82	0.61
	DM4_02	0.84		
	DM4_03	0.69		
DM5	DM5_01	0.91	0.91	0.78
	DM5_02	0.88		
	DM5_03	0.86		
DM6	DM6_01	0.62	0.88	0.65
	DM6_02	0.83		
	DM6_03	0.89		
DM7	DM7_01	0.87	0.82	0.61
	DM7_02	0.84		
	DM7_03	0.86		
DM8	DM8_01	0.61	0.87	0.69
	DM8_02	0.85		
	DM8_03	0.80		
DM9	DM9_01	0.85	0.85	0.58
	DM9_02	0.85		
	DM9_03	0.77		
	DM9_04	0.81		
DM10	DM10_01	0.59	0.87	0.70
	DM10_02	0.83		
	DM10_03	0.84		

Note: DM1 = Autonomous Choice to Use Mobile Devices; DM2 = Autonomy Within Digital Contexts; DM3 = Digital Literacy; DM4 = Individual Growth in Digital Contexts; DM5 = Digital Risk Awareness; DM6 = Support-Seeking Regarding Digital Problems; DM7 = Regulation of Negative Emotions in Digital Contexts; DM8 = Regulation of Impulses in Digital Contexts; DM9 = Respect Towards Others in Digital Contexts; DM10 = Digital Citizenship; CR = Composite Reliability; AVE = Average Variance Extracted.

**Table 4:** The correlations among digital maturity, digital native and its subdimensions.

Variables	1	2	3	4	5	6	Cronbach’s Alpha
DM ^a^	—						0.83
DN	0.25 ***	—					0.92
DN1	0.10 *	0.83 ***	—				0.88
DN2	0.25 ***	0.88 ***	0.70 ***	—			0.82
DN3	0.21 ***	0.87 ***	0.60 ***	0.67 ***	—		0.88
DN4	0.30 ***	0.79 ***	0.67 ***	0.57 ***	0.65 ***	—	0.79
Mean (SD)	3.64 (0.50)	3.22 (0.97)	2.85 (1.22)	3.24 (1.17)	3.20 (1.17)	3.58 (1.06)	

Note. * *p* < 0.05, *** *p* < 0.001. ^a^ DM was calculated from its 10 sub-dimensions according to the previous study. DN1 = Grow up with technology; DN2 = Comfortable with multitasking; DN3 = Reliant on graphics for communication; DN4 = Thrive on instant gratifications and rewards.

**Table 5:** The predictive validity of digital maturity on AI literacy.

Predictor		AI Literacy					
		Step 1		Step 2		Step 3	
		β	t-Score	β	t-Score	β	t-Score
Control	Gender	−0.126 **	−2.631	−0.118 **	−2.769	−0.143 ***	−3.510
	Grade	−0.062	−1.303	−0.054	−1.258	−0.056	−1.383
	Time spent on weekdays	0.122 *	2.156	0.075	1.481	0.083	1.721
	Time spent on weekends	−0.116 *	−2.049	−0.176 **	−3.457	−0.160 **	−3.298
Digital native				0.448 ***	10.305	0.372 ***	8.655
Digital maturity						0.285 ***	6.764
	F	3.763 **		24.987 ***		30.624 ***	
	R^2^	0.034		0.226		0.301	
	ΔR^2^	—		0.192		0.075	

Note. * *p* < 0.05, ** *p* < 0.01, *** *p* < 0.001.

**Table 6:** Reliability statistics and correlations among the digital maturity sub-domains for Study 2.

Variables	1	2	3	4	5	6	7	8	9	10	Cronbach’s Alpha
DM1 ^a^	—										0.69
DM2	−0.49 ***	—									0.84
DM3	−0.26 ***	0.39 ***	—								0.72
DM4	−0.19 ***	0.33 ***	0.49 ***	—							0.82
DM5	−0.07 *	0.24 ***	0.41 ***	0.59 ***	—						0.90
DM6	−0.06 *	0.13 ***	0.26 ***	0.46 ***	0.53 ***	—					0.87
DM7 ^a^	0.36 ***	-0.18 ***	−0.08 *	−0.10 **	−0.06 *	−0.16 ***	—				0.77
DM8 ^a^	0.41 ***	-0.23 ***	−0.13 ***	−0.07 *	0.00	−0.05	0.63 ***	—			0.78
DM9	−0.17 ***	0.28 ***	0.41 ***	0.41 ***	0.50 ***	0.37 ***	−0.10 **	−0.18 ***	—		0.85
DM10	−0.18 ***	0.22 ***	0.43 ***	0.43 ***	0.39 ***	0.33 ***	−0.24 ***	−0.26 ***	0.60 ***	—	0.85
Mean (SD)	3.88 (0.87)	3.26 (1.16)	3.22 (1.21)	3.65 (1.02)	4.18 (1.04)	3.51 (1.15)	3.92 (1.05)	4.02 (1.08)	3.58 (1.17)	2.99 (1.26)	

Note. * *p* < 0.05; ** *p* < 0.01; *** *p* < 0.001. ^a^ According to the original digital maturity scale, items for DM1, DM7, and DM8 were reversed coded and these sub-domains were calculated after reverse coding. DM1 = Autonomous Choice to Use Mobile Devices; DM2 = Autonomy Within Digital Contexts; DM3 = Digital Literacy; DM4 = Individual Growth in Digital Contexts; DM5 = Digital Risk Awareness; DM6 = Support-Seeking Regarding Digital Problems; DM7 = Regulation of Negative Emotions in Digital Contexts; DM8 = Regulation of Impulses in Digital Contexts; DM9 = Respect Towards Others in Digital Contexts; DM10 = Digital Citizenship.

**Table 7:** Model fits for the latent profile model.

Profiles	k	Log Likelihood	AIC	BIC	aBIC	Entropy	LMR	BLRT	Percentages
Profile 1	20	−15,485.851	31,011.702	31,110.370	31,046.848				
Profile 2	31	−14,749.678	29,561.355	29,714.291	29,615.832	0.831	=0.0061	<0.0001	0.257\0.743
Profile 3	42	−14,317.900	28,719.799	28,927.003	28,793.607	0.896	<0.0001	<0.0001	0.210\0.686\0.104
Profile 4	53	−13,977.652	28,061.305	28,322.776	28,154.443	0.848	=0.0078	<0.0001	0.076\0.091\0.427\0.405

Note. k = number of classes; AIC = Akaike Information Criterion; BIC = Bayesian Information Criterion; aBIC = adjusted Bayesian Information Criterion; LMR = Lo-Mendell-Rubin likelihood ratio test; BLRT = Bootstrap Likelihood Ratio Test.

**Table 8:** Means, standard deviations and one-way ANOVA results for three profiles.

Dimensions	Profile 1 (Unbalanced Low)	Profile 2 (Inconsistent Medium)	Profile 3 (Relatively High)	One-Way ANOVAs			
	N = 215 (21.0%)	N = 704 (68.6%)	N = 107 (10.4%)				
	Mean (SD)	Mean (SD)	Mean (SD)	df	F	*p*	Post Hoc
DM1 ^a^	4.07 (0.75)	3.95 (0.79)	3.04 (1.14)	2	64.271 ***	0.000	P1 = P2; P1 > P3 ***; P2 > P3 ***
DM2	2.67 (1.09)	3.34 (1.12)	3.98 (1.03)	2	55.180 ***	0.000	P1 < P2 ***; P1 < P3 ***; P2 < P3 ***
DM3	2.20 (0.95)	3.44 (1.13)	3.84 (1.03)	2	126.795 ***	0.000	P1 < P2 ***; P1 < P3 ***; P2 < P3 ***
DM4	2.55 (0.85)	3.92 (0.84)	4.13 (0.89)	2	234.055 ***	0.000	P1 < P2 ***; P1 < P3 ***; P2 < P3 *
DM5	2.63 (0.93)	4.59 (0.55)	4.57 (0.69)	2	741.922 ***	0.000	P1 < P2 ***; P1 < P3 ***; P2 = P3
DM6	2.47 (0.91)	3.75 (1.04)	4.00 (1.11)	2	144.203 ***	0.000	P1 < P2 ***; P1 < P3 ***; P2 < P3 *
DM7 ^a^	4.02 (0.88)	4.18 (0.77)	2.01 (0.93)	2	331.894 ***	0.000	P1 < P2 **; P1 > P3 ***; P2 > P3 ***
DM8 ^a^	4.07 (0.87)	4.34 (0.71)	1.83 (0.90)	2	495.974 ***	0.000	P1 < P2 ***; P1 > P3 ***; P2 > P3 ***
DM9	2.33 (0.88)	3.83 (1.01)	4.40 (0.78)	2	244.643 ***	0.000	P1 < P2 ***; P1 < P3 ***; P2 < P3 ***
DM10	1.98 (0.81)	3.10 (1.18)	4.30 (0.97)	2	171.539 ***	0.000	P1 < P2 ***; P1 < P3 ***; P2 < P3 ***
DM	2.91 (0.34)	3.87 (0.40)	3.61 (0.33)	2	535.685 ***	0.000	P1 < P2 ***; P1 < P3 ***; P2 > P3 ***

Note. * *p* < 0.017 (adjusted for multiple comparisons for all post hoc analyses), ** *p* < 0.01, *** *p* < 0.001. ^a^ According to the original digital maturity scale, items for DM1, DM7, and DM8 were reversed coded and these sub-domains were calculated after revise coding. DM = Digital maturity; DM1 = Autonomous Choice to Use Mobile Devices; DM2 = Autonomy Within Digital Contexts; DM3 = Digital Literacy; DM4 = Individual Growth in Digital Contexts; DM5 = Digital Risk Awareness; DM6 = Support-Seeking Regarding Digital Problems; DM7 = Regulation of Negative Emotions in Digital Contexts; DM8 = Regulation of Impulses in Digital Contexts; DM9 = Respect Towards Others in Digital Contexts; DM10 = Digital Citizenship.

## Data Availability

Data are available upon request from the authors.
